# Case conceptualization in child welfare: an underused resource to improve child, family, and provider outcomes

**DOI:** 10.3389/fpsyt.2023.1292690

**Published:** 2024-01-11

**Authors:** Jill R. McTavish, Angela McHolm, Anne Niec, Anna Marie Pietrantonio, Christine McKee, Harriet L. MacMillan

**Affiliations:** ^1^Department of Psychiatry and Behavioural Neurosciences, McMaster University, Hamilton, ON, Canada; ^2^Department of Pediatrics, McMaster University, Hamilton, ON, Canada

**Keywords:** child maltreatment, case conceptualization, child welfare, child mental health, intimate partner violence, family violence

## Abstract

Case conceptualization, formally known as case formulation, is one tool that assists in determining the best course of action for children and families experiencing family violence that has been under-utilized in child welfare. In this article we present a step-by-step case conceptualization process that considers the child welfare context. We then present a hypothetical case example of a 10-year-old child referred by a child welfare worker to evidence-based treatment for mental health and behavioural concerns. Mental health services are not helpful for the child and further consultation is enlisted. To more effectively guide intervention and treatment planning and ultimately improve outcomes for the child, we present case conceptualization as a process that incorporates relevant aspects of the child and family’s history and circumstance. We conclude with a succinct case conceptualization and treatment plan to show how the prognosis of the child can be improved when case conceptualization is employed.

## 1 Introduction

Children and families coming into contact with child welfare can represent some of the most complex cases for healthcare and social service providers to support, often due to interlocking socioecological factors, such as poverty, poor integration of services, high turnover of child welfare workers, poor family function, and intergenerational histories of trauma (1–14). Outcomes for children experiencing maltreatment are often framed as “gloomy” (15) and to date it is unclear if contact with child welfare improves child and family outcomes (9, 16). While a variety of evidence-based services for children and families exist, it can be challenging to determine the best course of action for treatment or support due to the complex needs of such children and families. Case conceptualization, formally known as case formulation, is one tool that can assist providers in determining the best course of action for supporting children and families that has been under-utilized in child welfare. As case conceptualization is an important clinical tool that can assist in improving client/patient outcomes (17) as well as provider outcomes (18, 19) [e.g., increased tolerance for uncertainty (20)], this commentary offers a succinct summary of the components of case conceptualization and gives examples of its application to child welfare. First, we discuss the importance of applying trauma- and violence- informed care principles to child welfare responses and the need for children involved in child welfare to undergo a comprehensive assessment in order to identify their needs and appropriate services. Second, definitions and important components of case conceptualizations are described. Third, a case example, ‘Rose’, is presented where evidence-based services are offered to a child; however, as the unique needs of the child and family were not first conceptualized in this case example, these services did not improve the child’s outcomes. The commentary then moves to a reconsideration of the case example to illustrate how to carefully tailor interventions to the unique circumstances of the child and family.

## 2 The importance of trauma- and violence-informed care

There are several important principles that inform safe and effective work with children and families involved with child welfare. Providers need to consider these principles at all phases of assessment and intervention, including when doing a case conceptualization. Some of these principles include the need for support and services to be evidence-based; tailored to the specific family/child including to the child’s age and stage of development; culturally sensitive; trauma-informed; comprehensive; and strengths-based (21). For example, principles of trauma-informed practice are increasingly incorporated into core competencies for healthcare and social service providers supporting children and families involved in child welfare (22–24). Trauma-informed care is a “whole system organizational change process which seeks to embed theoretically coherent models of practice across diverse settings and roles, including child welfare, family support, justice, mental health and education” (25). Trauma-informed principles have been incorporated into child welfare in a variety of ways, including through workforce development (e.g., training staff to understand the impact of trauma), service delivery (e.g., recognizing and integrating the child’s trauma history in case planning), and organizational change (e.g., increasing collaboration and information sharing) (25). There have been several critiques of trauma-informed care. For example, some authors suggest that it has a “relatively narrow definition of trauma that implicitly emphasizes violence between individuals,” it emphasizes “medical environments at the exclusion of others (e.g., legal systems, social services, educational systems, economic structures),” and it has an “implicit assumption that trauma affects everyone in the same way” (26). Some authors suggest that a lack of prioritization of these concerns has contributed to the pathologizing of individuals who have experienced interpersonal and structural violence (27).

In response to such criticisms, trauma- and violence-informed care was developed to draw attention to historic, intergenerational, and structural violence. Trauma- and violence-informed care “extends the trauma-informed care framework with the addition of ‘violence’ to emphasize the association between trauma and violence’’ (28, 29). Trauma- and violence-informed care addresses individual and broader systems-level aspects of the care encounter, including the environment of the care encounter (e.g., is the environment safe and welcoming?), the approach the provider uses (e.g., is the provider trained in how to respond safely to family violence?), and the provider’s response to the patient in the encounter (28). Trauma- and violence-informed care is intersectional, in that it considers an individual’s past and current experiences of trauma and how they intersect with past and current experiences of systematic or structural violence (e.g., racism, colonialism) (29, 30). Trauma- and violence-informed care is also attentive to the additive effects of trauma, in that it emphasizes how past, current, interpersonal, and structural violence can overlap to produce significant, negative health consequences (29, 30).

Principles of trauma- and violence-informed care can inform the approach to the case conceptualization, including 1) the recognition by providers of the high prevalence of family violence [defined as violence, abuse, conflict or neglect by a family member toward a family member that is associated with poor health (31)] and its impact on child well-being, 2) an emphasis by providers on the need to reduce possible victimization and future harm experienced by the child in their family and in their interactions with the provider; and 3) attention to physical, emotional, and cultural safety in all interactions between providers and clients (28). For example, for children involved in child welfare who are referred to mental health services, it is important to consider if past, current, and ongoing experiences of family violence may be influencing behavioural problems and if children have physical and psychological safety in their home environment in order to benefit from mental health support at the time. Many healthcare and social service providers are comfortable making referrals to counseling or psychiatric support for psychological and behavioural goals. However, this may not address the root causes of the child’s concerning behaviours and may not be in the best interest of the child if there is not physical and psychological safety within the family context (e.g., quality of relationships, family dynamics) at the time of service provision. For a case conceptualization and future treatment to be effective, children must first undergo a comprehensive assessment.

## 3 Comprehensive assessments for children involved in child welfare

The purpose of a comprehensive assessment is to identify key aspects of a child’s life and to consider evidence-based referrals that reflect the unique needs of the child, including their age and developmental stage (32). A trauma- and violence informed approach to comprehensive assessment would include attention to the environment within which the comprehensive assessment takes place (e.g., is the assessment environment safe and welcoming?), the provider’s approach to assessment before the encounter occurs (e.g., is the provider trained in how to provide a safe response to disclosures of family violence, are they culturally respectful, are they aware of the intersections between experiences of structural and interpersonal violence?), and the provider’s specific responses to children and families in the assessment (e.g., do they establish rapport with the child and family, do they consider their physical proximity when speaking with a child and family, do they ask the child’s preferred name, do they explain the limits of confidentiality in a developmentally appropriate way, are they non-judgmental, do they respect the inherent dignity of the child and family?) (28).

A comprehensive assessment should take into account the child’s home situation (e.g., description of people in the family, present living situation, extended kinship networks, any non-traditional familial relationships); their general development (e.g., cognitive, emotional, physical); their education (e.g., school, grades, teachers); their involvement with activities (e.g., recreation); their mental health, including questions about symptoms (including post-traumatic stress) and general functioning (e.g., well-being, sleep, physical health, peer relationships); their experiences of family violence and other adversities; and any other relevant aspects of their lives (e.g., substance use, sexual health) (28). With regard to asking about exposure to maltreatment, care should be taken not to overlap with the role of the child welfare worker; if the child discloses previously unreported information about suspected maltreatment, investigation of this information will typically be undertaken by the child welfare worker. Assessments should also consider relevant information about people of importance to the child (e.g., their siblings and their main caregivers’ personal, social, and health history; other caring adults; friendships). Assessments should enable providers to communicate with children alone, as well as time to observe them with their primary caregiver(s) (28). Gathering this information is necessary for developing an accurate case conceptualization (33). In many cases, referral to health services is informed by an assessment of an individual; however, especially when child welfare is involved, providers must understand family dynamics, including how the child interacts with their siblings and caregivers and the caregivers’ and siblings’ own histories. From this assessment, providers will be better able to assess the suitability of services and supports for the child in the context of their family.

## 4 Case conceptualization: definition and components

After a comprehensive assessment has been completed, healthcare and social service providers can build a case conceptualization incorporating all aspects of their interactions with the child or family, as well as any additional collateral information (for example, from teachers). Case conceptualization can be defined as “a clinical strategy for obtaining and organizing information about a client, explaining the client’s situation and maladaptive patterns, guiding and focusing treatment, anticipating challenges and barriers, and preparing for successful termination” (34). It is essentially a comprehensive map for treatment and support, which includes an overview of the child or family’s concerning symptoms and behaviours (diagnostic formulation); how these symptoms and behaviours came to be and are understood (clinical formulation); an understanding of the role of culture in the case (cultural formulation); and a treatment or support map that links specific child/caregiver problems with evidence-based services or supports (treatment formulation) (35, 36).

### 4.1 What symptoms and behaviours are the child or family struggling with (diagnostic assessment/formulation)?

When children and families present with behavioural and/or emotional problems, a diagnostic assessment/formulation is often prioritized in most healthcare and social service settings (including child welfare). This typically entails a description of the client’s presenting situation (e.g., concerning symptoms and behaviours) and any factors that prompt these concerns (35). A diagnostic assessment often leads to one or more diagnoses based on the Diagnostic and Statistical Manual of Mental Disorders (DSM). Many children involved in child welfare meet diagnostic criteria for a range of diagnoses, such as oppositional defiant disorder (ODD), conduct disorder, major depressive disorder, and/or reactive attachment disorder (37). Behaviours associated with these diagnoses are understandable given their experiences in unstable, chaotic, or unsafe home environments (38). In other instances, however, children exposed to family violence may not meet full diagnostic criteria (e.g., post-traumatic stress disorder) yet struggle significantly. In either situation, case conceptualization provides a much richer understanding of what contributes to, and maintains, a child’s emotional or behavioural difficulties. While diagnoses may be useful or necessary for accessing specific services, they are often limited to a summary of a child’s symptoms. A comprehensive case conceptualization subsumes any such diagnoses and better informs treatment planning, as it helps the provider shift from description to explanation and understanding.

For example, many children who experience child maltreatment, including exposure to intimate partner violence, have complex changes to brain development (e.g., impaired stress response), cognition (e.g., language delays, problems with concentration), behaviour (e.g., poor self-regulation, social withdrawal), mental health (e.g., depression, anxiety), relationships (e.g., poor understanding of social interactions), emotions (e.g., difficulty controlling emotions), and physical health (e.g., sleep disorders) (39–43). As referenced above, many maltreatment-related behavioural, emotional, and relational changes that children experience overlap with other common diagnoses, such as ODD (see Table 1). For example, the National Institute for Health and Care Excellence details over 70 indicators of child maltreatment. Behavioural indicators like “markedly oppositional behaviour,” emotional indicators like “repeated, extreme or sustained emotional responses,” and relationship indicators like “coercive controlling behaviour towards parents or carers” are closely related to the diagnostic criteria for ODD. Signs and symptoms of other disorders also overlap with maltreatment symptoms. For example, signs of attention deficit hyperactivity disorder (ADHD) (e.g., agitation, poor self-esteem, difficulties concentrating, and difficulties with work, school and sleep) are common in children who have experienced maltreatment (32, 38, 46, 47). When maltreatment-related symptoms are not recognized or treated inappropriately—for example, if symptoms are inappropriately treated with pharmacological interventions—the relational injuries that are underlying the child’s symptoms are not addressed (38). Following from a comprehensive assessment, such behaviours can be better understood as traumatic stress reactions given exposures to family violence. Developmental trauma disorder has long been proposed by clinicians and researchers as a way to capture the clinical presentation of children who have been exposed to chronic interpersonal trauma (48–50).

**Table 1:** Comparing indicators of maltreatment (44) to ODD diagnostic criteria (45).

Area of child development	Examples of signs of child maltreatment (44)	Examples of diagnostic criteria for ODD (45)
Behaviour	“Markedly oppositional behaviour” “Aggressive, oppositional”	“Often actively defies or refuses to comply with requests from authority figures or with rules”
Emotions	“Extreme distress” “Lack of ability to understand and recognize emotions” “Repeated, extreme or sustained emotional responses” “Anger or frustration expressed as a temper tantrum in a school-aged child”	“Is often touchy or easily annoyed” “Often loses temper” “Is often angry and resentful”
Relationships	“Coercive controlling behaviour towards parents or carers”	“Often argues with authority figures or, for children and adolescents, with adults”

In addition, child welfare workers may become aware of parental mental health concerns with symptoms consistent with disorders, such as borderline personality disorder or narcissistic personality disorder. While understanding a person’s diagnosis may be helpful, building a treatment plan from a diagnosis only is an example of “backward reasoning,” which involves creating a hypothesis about treatment and then scanning to find supporting data (51). This reasoning involves a narrowing of options and does not effectively address the unique aspects of the client’s life. A more helpful strategy in case planning is to use “forward reasoning,” or to use specific incidents presented by the client (or observed by the practitioner) to develop hypotheses (51). For example, if during the course of the comprehensive assessment, it was observed that a mother and her children were reluctant to speak in front of a father who appeared agitated and directed most of the conversation, and the clinician was aware of a history of reports to child welfare for intimate partner violence (IPV), the clinician might surmise that various safety strategies were needed early on when working with this family. The clinician would need to prioritize assessing the safety of the mother and the children before specific clinical services or supports could be offered. In this example, the clinician might also assume that couples’ therapy was contraindicated given potential safety concerns arising from past (and potentially current) IPV (52).

### 4.2 Why did it happen to this child or family (clinical assessment/formulation)?

An essential component of a case conceptualization is a clinical formulation, which investigates what happened to this child to explain the “why” of the child or family’s presenting concerns, given their history or current life stressors. Attention to the *why* of behaviour is a principle of trauma- and violence-informed care, as given the high prevalence of family violence it is important to consider how child and parent behaviours make sense given their potential history of family violence (29). Clinical formulations provide an explanation of a client’s behaviour, usually through a particular theoretical lens, such as through a bio-psycho-social-spiritual lens or a cognitive-behavioural lens. The discussion below does not represent an exhaustive list of the theoretical frameworks/lenses that can inform a conceptualization but provides examples that may be helpful to characterize how case conceptualization must attend to both broad (structural) concerns as well as individual concerns. While not discussed in the present manuscript, other theoretical lenses for case conceptualization are available, such as the Attachment, Self-Regulation, Competency (ARC) model, are available (33, 36, 51, 53, 54).

A socioecological (also sometimes referred to as a critical ecological or ecological) lens is especially relevant when undertaking a case conceptualization for children and families involved in child welfare, as this model is commonly used in violence prevention research to outline the range of factors influencing risk and prevention of violence (55). This model, for example, attends to risk and protective factors at the societal level (e.g., societal norms regarding physical punishment); community level (e.g., availability of coordinated services for children and families); institutional level (e.g., level of support from child welfare workers); relationship-level (e.g., caregiver-child attunement); and individual level (e.g., caregiver mental health concerns). For example, a recent systematic scoping review found that youth (15+) and adults of colour accessing sexual assault services experienced many barriers to care, including a lack of access to diverse staff at sexual assault services and experiences of discrimination/racism from white service providers (56). Absence of culturally safe services at the community level is a risk to children and families, in terms of effective treatment planning; providers also have an opportunity to advocate for meaningful services for children, youth, and families in these circumstances. The socioecological model can also help to balance individual concerns (e.g., caregiver mental health concerns) and structural concerns (e.g., housing instability, experiences of racism). As outlined in the literature, child welfare scholars are often split across the individual-structural divide (i.e., authors tend to exclusively adhere to one theoretical lens or the other), which offers only a partial assessment of client problems and capabilities (57). For example, in discussing children’s experiences of family violence some authors focus exclusively on structural determinants, such as racist legislation or inadequate government funding for programming, without discussing the impact of parental mental health concerns on children whereas other authors focus exclusively on programs to address parenting behaviours without attending to structural factors affecting parents. Both lenses are required for effective case conceptualization for child welfare cases.

While individual providers may not be able to effect immediate change in factors at the societal, community, or institutional level, it is important for providers to have awareness of these factors. At the individual/relationship level, or in direct clinical work with children and families, the bio-psycho-social-spiritual model remains an important model for case conceptualization as it is largely atheoretical (meaning it can be used across diverse professions that are applying different theoretical lenses) (51). It is also broad enough to cover biological concerns (the domain of healthcare providers such as physicians and nurses), psychological concerns (the domain of psychologists and psychotherapists), and social domains (the domain of teachers, recreational professionals, and advocates), as well as the intersections between these domains. The bio-psycho-social-spiritual model can help lessen the tendency for case conceptualizations to be overly biomedical or overly psychological. As such the bio-psycho-social-spiritual model may represent a useful framework for undertaking comprehensive assessments in child welfare. For example, children involved with child welfare tend to receive more pharmacological interventions than their peers (58, 59), even though in many cases it is not recommended or is even contraindicated. Also, as discussed above, many providers feel comfortable making referrals to mental health services but overlook consideration of children’s need for positive social supports, such as recreational camps or tutoring.

Socioecological and bio-psycho-social-spiritual factors can be considered across the “5 Ps,” or across precipitants, perpetuants, predisposing factors, and protective factors that inform the client’s presenting pattern. The 5 Ps are commonly used in clinical formulations to account for the client’s pattern of functioning, or when considering (in)flexible and (in)effective ways that the client is thinking, perceiving, and acting. For example:

- Presenting factors attend to the child’s attentional and behavioural issues, such as physical aggression toward peers, distractibility, and resistance to following instructions.
- Precipitant factors include triggers that bring about the client’s presenting concern. For example, if a child’s behavioural problems at school were triggered by peers yelling, this would give us important information about the child’s vulnerability to loud noises or would cause us to wonder if the child was exposed to loud noises in their environment (e.g., yelling in the home).
- Perpetuating factors maintain the client’s presentation. For example, if a child’s teacher lacked skills for understanding how trauma can manifest in concerning behaviours, they may not have a compassionate and understanding response to the child’s behaviours, unintentionally helping to maintain the behaviour. School can be an important resource for children with experiences of violence, as children may look to schools as a “safe haven” free from violence, a predictable setting in comparison to their “chaotic” and “unpredictable” homelife, and a place where it is possible to develop safe and supportive relationships with teachers and peers (60).
- Predisposing factors include bio-psycho-social-spiritual factors that contribute to the client’s presentation. For example, if a child is aware of past or current IPV in their home or had experienced harsh discipline from their caregivers, this could have significant negative effects on their development, including changes in brain development, a decreased capacity for emotional regulation, and increased threat sensitivity (61, 62).
- Protective factors include bio-psycho-social-spiritual factors that lead to adaptive presentation and functioning (e.g., curiosity, spirituality, high-quality daycare or school environments). For example, a teacher’s care and concern for a child could be a protective factor. Other significant resilience factors have been found for children experiencing maltreatment, such as family and peer factors (e.g., maternal sensitivity, close mother–child relationship, friendship, and social support) (8).

Clinical formulations can draw on a variety of theories to understand the client’s unique pattern of functioning, such as theories about stages of change, psychodynamic theories about personality development, theories about family functioning, and considerations of attachment theory (36). For example, it is important to consider the client’s readiness for treatment, or to consider the stages of change, and to tailor treatment accordingly (63). Consider a caregiver who has been referred to an evidence-based parenting program for substantiated physical abuse of their child. The treatment trajectory and prognosis would be very different for this caregiver depending on if they were in the precontemplation phase of change (they do not consider their behaviour to be a problem and do not feel they need to change) as compared to the preparation phase of change (they have made a commitment to change their behaviour, which they consider problematic, and they may even have identified steps towards change). Some child welfare interventions have begun to acknowledge stages of changes by modifying interventions to explicitly address client readiness and stages of change (64); this represents a generalized strategy that may or may not be needed depending on the specific client.

In addition to considerations of stages of change, it is important to consider hierarchies of power in families, such as gender and intergenerational power, especially when family violence is a concern. Researchers attending to hierarchies of power discuss the need to address safety and protection of children; empowerment and safety of women and 2SLGBTQ+ persons; and responsibility and accountability for those using violence in their relationship (65). When there are concerns about family violence in the home, a provider’s attention to hierarchies of power can help them to transparently suggest avenues for action when supports and services are not working to address safety in the home. For example, Humphreys (65) has outlined avenues of action when there is a conflict in needs between children experiencing safety concerns in the home, women experiencing IPV, and men using violence in relationships:

For example, should there be a dilemma between the principle of child safety and that of the empowerment and safety of women, which even after high level support is unable to be addressed, then the safety of children remains paramount due to their level of vulnerability. Similarly, if there is a conflict of interest or resourcing pressures, the safety and empowerment of women needs to be placed as a priority over potential work with men.

For children involved in child welfare who are experiencing current maltreatment, including exposure to IPV, treatment planning might first involve addressing safety in the home, for example, by working to increase caregivers’ supportive and safe behaviours. This could entail involving caregivers in an evidence-based parenting program with motivational interviewing components (66). Parallel work could be done with a caregiver who is exposed to IPV to assess their safety and refer them to evidence-based resources to address their past or current experiences of IPV [e.g., structured advocacy interventions or support for any symptoms resultant from past experiences of violence (52)]. This work may be important to do before a child is referred to evidence-based services for their emotional and behavioural problems, as these concerns may be the direct result of lack of safety in the home. If work to increase safety in the home is not successful, and safety continues to be a concern for the involved children, additional approaches will need to be considered to prioritize child safety, including the potential for high-quality out-of-home care placement, such as kinship or foster care.

### 4.3 What role does culture play (cultural formulation)?

A cultural formulation answers the question “what role does culture play” by analyzing salient cultural factors, such as level of acculturation and stress (51). Effective cultural formulation is essential in child welfare, especially given the overrepresentation of certain racial, ethnic, and cultural groups, such as Black and Indigenous families (67, 68). Cultural considerations can be considered across the bio-psycho-social-spiritual framework, including:

- Biological concerns (e.g., any particular health concerns common in the family’s history; any biological impacts of intergenerational or racial trauma, such as worsening of chronic illness),
- Psychological concerns [e.g., social or cultural identity; cultural explanations, or culturally influenced beliefs about the client’s presenting concern (51)],
- Social concerns [e.g., cultural stress; acculturation, or level of adaptation to dominant culture; any culturally-influenced stress or protective factors; a history of intergenerational or racial trauma; cultural expectations of parenting (69)], and
- Spiritual concerns (e.g., spirituality as a protective factor and/or as a source of cultural/racial persecution).

Using a trauma- and violence-informed care lens, cultural awareness can include attention to ways to increase cultural safety in the environment, approach, and provider response (28). For example, in terms of the environment, some Indigenous clients may appreciate access to an Elder in the service organization or through a referral (70). In terms of the approach, it is important for providers to be aware of a client’s potential experiences of social or cultural violence, discrimination, stigmatization, or oppression (e.g., feeling misunderstood; misjudged related to social/cultural identity; direct experiences of discrimination, stigma, oppression, exclusion, ostracization, or being devalued; experiences of microaggressions; difficulties assimilating). This might involve a provider undertaking training about historical violence, for example, as summarized in the Truth and Reconciliation Commission of Canada Calls to Action (71). It can be desirable for providers to make efforts to increase working relationships with social and cultural community leaders and organizations, to increase the provider’s own awareness of culturally appropriate and available services in the community (72). Such awareness will enable the provider to facilitate and tailor referrals to appropriate services when indicated. In terms of the provider response in the care encounter, it is important for providers to explore and understand the client’s cultural explanation, or culturally influenced beliefs about the client’s presenting concern (51). Understanding cultural explanations of the client’s presentation is an important part of treatment planning (73), as divergent client and provider understanding of the 5 Ps can lead to treatment ‘poorness of fit’ between provider and client goals, as well as poor treatment prognosis (34).

### 4.4 How can what happened and its impact be addressed (treatment formulation)?

A treatment formulation answers the question “how can it be changed” by specifying a map for treatment planning. Treatment formulations address the focus, goals, strategies, and interventions of treatment, as well as treatment obstacles and prognosis. Treatment focus addresses the direction of treatment; it is akin to the metaphor of a map, which shows the best route to achieve a desirable treatment outcome (51). The map for a bio-psycho-social-spiritual approach addresses concerning situations that prompted or were exacerbated by biological, psychological, social, or spiritual vulnerabilities. For children involved in child welfare who are experiencing ongoing safety concerns, the treatment focus may involve a continual reorientation to safety in the home and services and supports to address caregivers’ biological, psychological, social, and spiritual vulnerabilities. Treatment goals are realistic, measurable, and achievable; a goal can represent the final destination on the map or can involve small stops on the map as the client moves towards the final treatment outcome. An example of a goal in a parenting class would be to observe an increase in parental nurturing and responsive behaviours, such as praising positive child behaviours, reflecting appropriate speech of children, or letting children lead conversations. Even in situations where treatment is mandated, goals should be mutually agreed upon by the provider and client. This may involve engagement to first address how services can meet both provider and client goals. Goals can be short-term (e.g., symptom reduction, increased adaptive functioning) or longer-term (e.g., pattern change) (51).

Treatment strategy refers to “the action plan for focusing specific interventions to achieve a more adaptive pattern” (51); it is akin to selecting the best route and vehicle to achieve the treatment goal. A *treatment strategy* involves the selection of appropriate *treatment interventions*, or actions designed to positively impact the client’s issue or problem. For example, where there is safety in the relationship between caregivers (e.g., no current or recent concerns about IPV), parent–child interaction therapy is an evidence-based intervention for children with externalizing problems who have a history of physical abuse or neglect. Usually this involves specific treatment interventions that teach the importance of child-directed interactions, including specific skills for caregivers to do more of (e.g., praise, reflect appropriate emotional response, imitate appropriate play, describe appropriate behaviour, and enthusiasm) (74).

Treatment formulation also considers treatment obstacles and prognosis. One test of an effective case conceptualization is its ability to predict the most likely obstacles and challenges (51), such as difficulties with engagement, ambivalence, and alliance. For example, as discussed above, it is important to assess the client’s readiness for change and to choose appropriate engagement strategies based on their level of engagement. It is also important to assess for practical barriers to engagement, such as lack of transportation, and to problem solve with clients. Second, many clients have ambivalence about services and treatments, with part of them moving towards change and another part of them resisting change. Practitioners need strategies to support clients in moving towards change, such as those offered by motivational interviewing (75). Third, while a working alliance is a consistent mediator of change (76, 77), including in contexts where treatment is mandated [for example, by child welfare (78)], difficulties with alliance between providers and clients should be anticipated and problem-solved in child welfare work. Improving alliance between providers and clients may involve provider skills (e.g., their ability to have conversations that repair alliance); alliance is also impacted by structural concerns [e.g., child welfare workers’ alliance with clients is better when there are dedicated family coordinators with low caseloads (79)].

Long-term outcomes for a child involved with child welfare can be linked to a number of factors, including child safety (recurrence of maltreatment, serious injuries/deaths), child well-being (school performance, including grade level and graduation; child behaviour; criminal justice involvement), permanence (placement rate, moves in care, time to achieving permanent placement); and family and community support (family moves, parenting capacity, ethno-cultural placement matching) (80). Even when there are intertwined factors that suggest a poor prognosis for a child, providers still have a meaningful opportunity to improve safety in the child’s home in order to hopefully prevent future experiences of maltreatment and more serious behavioural concerns.

## 5 A case example

Below we present a case example of a child who was referred for mental health services by a child welfare worker, which is a common type of service referral made by child welfare workers.

Rose is a 10-year-old girl who was referred by a child welfare worker for treatment of mental health and behavioural concerns. Child welfare was initially contacted by a school principal after a teacher raised concerns about Rose’s hygiene, inadequate lunches, and excessive sleepiness at school. The school personnel had tried to address these concerns with the parents, but the parents were not responsive and after raising these concerns Rose was increasingly absent from school. There were significant concerns about Rose’s behaviour at school, including physical aggression toward peers, distractibility, and resistance to following instructions; she was also easily startled and became irritable when class discussion was happening. The teacher questioned whether Rose had ADHD. Child welfare had been involved with the family for approximately six months after Rose was increasingly absent from school without explanation. Rose has five other siblings who were also struggling. Child welfare had made referrals for services. Rose’s parents were encouraged to have Rose seen by her family physician regarding her attentional and behavioural problems and she was referred for individual counseling to address her behaviours. The counselor working with Rose expressed concerns for Rose’s development including difficulty identifying and communicating her feelings and needs, and a tendency to portray herself negatively. For example, when asked why she thought she had come to see the provider, Rose responded “I was bad.” After 12 sessions with the counselor, Rose’s functioning and behavioural concerns at school had not improved. The counselor was concerned and sought supervision for the case. Individual sessions had focused on helping Rose to identify and communicate her needs and cognitively restructure her maladaptive thoughts and beliefs. Although such intervention strategies can be helpful for many children with similar emotional and behavioural concerns, they were not effective for Rose.

This case example illustrates how case prognosis can be poor for clients when key elements of the case are missing from the case conceptualization, such as sibling and family dynamics. When key elements of the case are considered, the likelihood of a positive treatment outcome is increased. Additional aspects that are important to consider in Rose’s case are discussed below.

Here is an example of additional information that was revealed about Rose’s living situation during a comprehensive assessment with Rose and her caregivers:

- *Description of people in the family, present living situation, extended kinship networks, any non-traditional familial relationships*: Rose’s father, Jake, identifies as Canadian/White with British heritage and her mother, Jalen, identifies as Southeast Asian. Jalen and Jake have 6 children in total between the ages of 3 and 12. The older children, including Rose, identify as Canadian/White. The family lives in a small, 3-bedroom apartment in the city and are isolated from family and community support due to frequent moves. Jake previously worked in construction but lost his job 3 years ago and has been working part-time as a painter since then. Jalen works part-time as a server in a local restaurant.
- *Developmental history*: Rose was born a month prematurely and had some early language delay that subsequently improved after beginning daycare.
- Education: Rose is academically behind her peers in terms of grades and social skills. Her teachers reported that she is quiet and “spaced out” but that she reacts violently towards peers (“goes from 0 to 100”) when she experiences loud noises by peers or in the classroom. Her teachers struggle to connect with Rose; they worry that Rose never smiles. They also reported that she misses a lot of school.
- *Involvement with activities*: Rose was not currently involved in any extra-curricular activities. Her parents have indicated that they do not have the resources and also identified they do not have time to take Rose to after-school activities.
- *Emotional/behavioural/psychological functioning*: Rose was identified as having difficulty communicating her feelings and a tendency to portray her role in family interactions negatively (“I was bad”). Concerns were identified that Rose exhibited symptoms of post-traumatic stress including hypervigilance, intrusive thoughts, sleep disturbances, emotional dysregulation, and dissociation (“spacing out”). During an individual interview with Rose, she disclosed fear about her mother’s well-being, as well as experiences of emotional abuse and neglect.
- *Information about caregivers and siblings*: The family had been referred to child welfare in the past because of concerns about IPV and Jake’s threats to hit his wife and the children. The family was considered a flight risk by child welfare, as they have had a history of moving when child welfare has become more intrusive. Three of Rose’s siblings have been referred to a pediatrician or a mental health professional, including psychiatrists, in the past and several have been diagnosed with ODD and ADHD and prescribed psychotropic medications. During the comprehensive assessment, Jake insisted that the child welfare worker had no authority in his family and that he has been doing his best for them. In a separate meeting with Rose’s mother, Jalen, she appeared to experience difficulty communicating about family relationships, and was vague in her responses. Jalen reported that Jake sometimes got “out of hand” but that he was doing the best for the family. Jalen disclosed being slapped across the face by Jake as recently as a year ago when Jake was intoxicated and expressed worry about Jake’s increasing reliance on alcohol since his job loss 3 years ago. Jake has previously been involved with psychiatric services, however he refused to provide additional details about the nature of these services (including any diagnoses). During an individual interview, Jake acknowledged feeling anxious and depressed about finances; he also discussed how he used alcohol to cope with stress. Jake also acknowledged previously hitting Jalen several times and expressed regret and a desire to never hit her again.
- *Observing Rose with her caregivers*: Rose, her siblings, and mother/father were observed together. In this meeting, Rose, her siblings, and the mother were all silent and reluctant to speak even when spoken to directly. Jake appeared agitated and directed most of the conversation, glaring at his children and wife and speaking for them when they were called upon by the provider. During this meeting Jake expressed sentiments of male privilege, such as discussing women’s role to listen and serve.

These additional details will be integrated into a clinical formulation of the case, as discussed below.

### 5.1 Clinical example revisited

Below we present a brief clinical formulation and treatment formulation statement for Rose based on the information from the comprehensive assessment.

Based on parent and teacher reports, Rose has a longstanding history of difficulties in functioning at school and within peer relationships. Her challenges with attention in the classroom were attributed by her pediatrician to a diagnosis of ADHD, and her aggressive interactions with peers led to a diagnosis of ODD. Even though Rose’s symptoms are reflected in these diagnoses, there are many aspects that are overlooked without a trauma- and violence-informed case conceptualization. Through a comprehensive assessment that provided Rose the opportunity to discuss her family relationships, Rose disclosed a history of longstanding fear about her mother’s wellbeing, as well as chronic emotional abuse and neglect from both parents. In individual interviews with each parent, Jalen spoke about being slapped across the face by Jake as recently as a year ago when Jake was intoxicated. She spoke about his problem with alcohol use that had increased following the loss of a full-time job 3 years earlier. In his interview, Jake initially denied any problems in the family, but subsequently spoke about feeling increasingly anxious and depressed about finances and using alcohol to cope. He acknowledged hitting Jalen on several occasions, and wanting to ensure this did not happen again, but had not told anyone including his family physician about his abusive behaviour.

To develop an understanding of Rose’s issues, we need to consider her presenting symptoms in the context of her life experiences, including her relationships with caregivers. Rose has been exposed over many years to IPV and has experienced emotional abuse and neglect. Her symptoms of ADHD and ODD, as well as physical problems, including difficulty sleeping, can best be understood by considering the principles of trauma- and violence-informed care. Rose’s presenting symptoms can be understood through the lens of complex trauma, which refers to experiences of multiple, traumatic events (e.g., exposure to maltreatment, loss of family relationships; inconsistent parenting etc.), specifically in the context of the child’s primary caregiving relationship(s). A child’s emotional well-being depends largely upon having a relationship with a caregiver who serves as a source of safety, security, and support. When a caregiver is perceived to be inconsistent, absent, or frightening during the early years of a child’s life, the child’s ability to tolerate and manage strong emotions, deal with daily stressors, develop self-confidence, and learn the foundations of relationships is compromised. When the parent is the source of the threat or maltreatment, then children learn to mistrust others and the world. As such, their capacity to develop emotional regulation skills is compromised, as the caregiver is not available to assist the child in regulating their emotions.

Children who experience maltreatment, including IPV exposure, can feel that they have to be on guard all the time; they often manifest this hypervigilance through distraction at school, impulsivity and aggression. Given Rose’s family life and experiences, her symptoms can be understood as adaptive responses to a maladaptive home environment. As such, a key priority in treatment planning involves preventing ongoing exposure to these experiences.

The foundation for Rose’s recovery is first and foremost a stable, nurturing parenting environment that is responsive to her needs and can provide her with opportunities for growth and development in a physically and psychologically safe context. While Rose requires ongoing support and assistance, it is essential that the trauma to which she has been exposed is prevented from recurring.

To address Rose’s needs, it is essential that the family needs are also considered and addressed. Specifically, it will be important to engage the parents in services to address their own respective experiences of trauma and mental health issues. Additionally, it will be important to work with the parents to increase their capacity to interact with Rose and her siblings in ways that are safe and supportive and avoid threatening or harmful behaviours. To do so, the parents need to acknowledge their role and responsibility in the harm suffered by their children, and there needs to be ongoing assessment of the parenting that Rose and her siblings are receiving. Connecting the parents to an in-home evidenced-based trauma and attachment-informed parenting program would be beneficial. This can only occur however, if Jake refrains from any further violence toward Jalen, and engages with substance use treatment and ongoing involvement with child welfare to assess the risk of violence in the home. Given the history of IPV, it is important for Jalen to have access to ongoing assessment of her safety and resources and support to address her past experiences of IPV. Given Jake’s patriarchal assumptions about men’s and women’s roles, it would be important to assess if these sentiments mirror his or Jalen’s understanding of gender and how this does or does not relate to their cultural heritage. It would also be important to assess if Jalen experiences cultural barriers to seeking help.

If safety is achieved in the family, work to support Rose’s individual and specific needs (e.g., traumatic stress symptom reduction, trauma processing, developing skills to manage difficult feelings, etc.) and experiences (including at school) may be indicated, including support to increase her tolerance of loud noises and work to support her success in classwork (e.g., tutoring).

If work to increase safety in the home is not successful, and safety continues to be a concern for Rose and her siblings, additional services need to be considered to prioritize child safety, including the potential for high-quality out-of-home care placement, such as kinship or foster care.

Children and family benefit from social and community supports. Rose’s family’s low socioeconomic status and social identity likely also influence their engagement and access to support and services. Given the family’s relative social isolation, consideration should be given to increasing the family’s opportunities to connect to additional supports and activities in the community which can be protective and increase safety for all members of the family. For instance, children’s successful participation in social/recreational activities can be beneficial and therapeutic as children are provided opportunities to develop their skills and talents, experience membership, a positive self-concept and self-esteem. Additionally, having access to healthcare providers, ideally in a community-based healthcare team that is trauma- and violence-informed, would be supportive not only to Rose, but also to her siblings and her parents. School can also be an important resource for children with experiences of violence. Rose’s teachers have identified their concerns for Rose. The school’s interest and care for Rose can be protective. It will be important for the school to have some understanding of Rose’s experience of trauma and how to support her within the context of trauma- and violence-informed care. Additionally, it is important for the parents’ relationship with the school to be facilitated and supported. This will likely require intervention (advocacy, psycho-education) from the family’s primary service provider.

Finally, access to services and supports for the family must be considered in the context of larger structural issues and barriers. For instance, this family has experienced inadequate and inconsistent housing and struggles financially. It will be essential to work with the family to ensure they have access to financial resources they are entitled to and to support and advocate for access to adequate and safe housing and transit/transportation. To be effective, service providers will need to develop collaborative working relationships with the parents. If these structural barriers are not addressed, the likelihood that individual interventions outlined above will be effective are significantly reduced.

### 5.2 Case commentary

In this example, the case conceptualization guides treatment planning and implementation, in order to prioritize creating safety in the family environment before addressing Rose’s mental health symptoms. The prioritization of safety is an essential component of treatment for clients with experiences of complex trauma (81). The case prognosis could be considered poor given the following factors: the family has been involved repeatedly with child welfare with the most recent opening occurring 6 months ago; Rose has poor school performance and struggles with post-traumatic stress; the family has a tendency to move; and the father does not appear ready to examine his abusive interactions and potential IPV in the home. However, progress is more likely to occur when experiences of family violence are understood and prioritized in treatment planning. Understanding principles of trauma- and violence-informed care can also help to put Rose’s physical, emotional, and social symptoms into context: they make sense considering her chronic experiences of maltreatment. Case conceptualization, including a bio-psycho-social-spiritual assessment, offers a strategy and rationale for sequencing interventions. By outlining the case conceptualization and treatment plan we make explicit our assumptions as providers, which we can evaluate in the future, based on treatment progress or barriers. For example, if the family’s housing issues are addressed, Jake’s substance use problems are managed appropriately, safety is achieved in the home (i.e., absence of IPV or child maltreatment), and Rose’s symptoms still persist, then we can begin to formulate additional factors that may be influencing her presentation.

## 6 Conclusion

In this paper we have shown how principles of trauma- and violence-informed care, comprehensive assessment, and case conceptualization can guide treatment planning and implementation, in order to best assess, address, and prioritize biological, psychological, social, and spiritual elements of the child and family. Case conceptualization in child welfare is warranted because of the inherent complexity of presenting cases. Further work in this area could evaluate ideal theoretical frameworks for developing effective and clinically useful case conceptualizations, as well as the potential benefits of interdisciplinary case conceptualization.

## Ethics statement

Written informed consent was not obtained from the individual(s) for the publication of any potentially identifiable images or data included in this article because while this paper is written like a case report, it is a hypothetical case based on common cases presented in the literature.

## Author contributions

JRM: Conceptualization, Writing – original draft, Writing – review & editing. AM: Conceptualization, Writing – review & editing. AN: Conceptualization, Writing – review & editing. AP: Conceptualization, Writing – review & editing. CM: Conceptualization, Writing – review & editing. HLM: Conceptualization, Resources, Writing – review & editing.
